# Peripheral endocannabinoids in major depressive disorder and alcohol use disorder: a systematic review

**DOI:** 10.1186/s12888-024-05986-8

**Published:** 2024-08-08

**Authors:** J.J. Fuentes, J. Mayans, M. Guarro, I. Canosa, J.I. Mestre-Pintó, F. Fonseca, M. Torrens

**Affiliations:** 1 Mental Health Institute, Hospital del Mar, Barcelona, Spain; 2 Department of Psychiatry and Forensic Medicine, Universitat Autònoma de Barcelona (UAB), Cerdanyola del Vallés, Spain; 3 Department of Psychiatry, Parc Sanitari Sant Joan de Déu, Sant Boi de Llobregat, Spain; 4 Hospital del Mar Research Institute, Barcelona, Spain; 5 Department of Medicine and Life Sciences, Universitat Pompeu Fabra, Barcelona, Spain

**Keywords:** Alcohol use disorder, Major depressive disorder, Endocannabinoids, Biomarkers

## Abstract

**Background:**

Major Depressive Disorder (MDD) and Alcohol Use Disorder (AUD) are two high-prevalent conditions where the Endocannabinoid system (ECS) is believed to play an important role. The ECS regulates how different neurotransmitters interact in both disorders, which is crucial for controlling emotions and responses to stress and reward stimuli. Measuring peripheral endocannabinoids (eCBs) in human serum and plasma can help overcome the limitations of detecting endocannabinoid levels in the brain. This systematic review aims to identify levels of peripheral eCBs in patients with MDD and/or AUD and find eCBs to use as diagnostic, prognostic biomarkers, and potential therapeutic targets.

**Methods:**

We conducted a systematic literature search according to the Preferred Reporting Items for Systematic Reviews and Meta-Analysis (PRISMA) guidelines from the earliest manuscript until October 22, 2023, in three electronic databases. We included studies of human adults who had a current diagnosis of AUD and/or MDD and evaluated plasma or serum endocannabinoids. We carefully considered known variables that may affect endocannabinoid levels.

**Results:**

We included 17 articles in this systematic review, which measured peripheral eCBs in 170 AUD and 359 MDD patients. Stressors increase peripheral 2-arachidonyl-glycerol (2-AG) concentrations, and 2-AG may be a particular feature of depression severity and chronicity. Anxiety symptoms are negatively correlated with anandamide (AEA) concentrations, and AEA significantly increases during early abstinence in AUD. Studies suggest a negative correlation between Oleoylethanolamide (OEA) and length of abstinence in AUD patients. They also show a significant negative correlation between peripheral levels of AEA and OEA and fatty acid amide hydrolase (FAAH) activity. Eicosapentaenoylethanolamide (EPEA) is correlated to clinical remission rates in depression. Included studies show known variables such as gender, chronicity, symptom severity, comorbid psychiatric symptoms, length of abstinence in the case of AUD, and stress-inducibility that can affect peripheral eCBs.

**Conclusions:**

This systematic review highlights the important role that the ECS plays in MDD and AUD. Peripheral eCBs appear to be useful biomarkers for these disorders, and further research may identify potential therapeutic targets. Using accessible biological samples such as blood in well-designed clinical studies is crucial to develop novel therapies for these disorders.

**Supplementary Information:**

The online version contains supplementary material available at 10.1186/s12888-024-05986-8.

## Background

Major Depressive Disorder (MDD) and Alcohol Use Disorder (AUD) are highly prevalent mental health conditions, and they tend to co-occur more frequently than one would expect by chance [1]. MDD is the most prevalent psychiatric comorbidity among patients with AUD [2, 3]. These two disorders are reciprocal risk factors, and patients with both conditions tend to experience more severe symptoms, higher psychosocial needs, an increased risk of suicidal behaviour [4] and require more healthcare resources [5]. However, identifying MDD in people who also consume alcohol could be challenging as alcohol consumption and withdrawal symptoms may mimic depressive symptoms [6]. Besides, it is important to differentiate between primary and induced major depressive disorder [7], as they differ in terms of prognosis, risk of relapse [8], and response to antidepressants [9].

The endocannabinoid system (ECS) moderates interactions among various neurotransmitters, which is crucial in regulating emotions [10], including the extinction of aversive memories and anxiety [11]. It also affects behavioural responses to stress and reward stimuli [12, 13], neuroinflammation, and neuroplasticity [14].

There is an increasing amount of evidence indicating that the ECS plays a crucial role in the pathogenesis of depressive disorders [15–17]. Chronic cannabinoid type 1 receptors (CB1R) blockade in animals induces anhedonia-like reactions [18] and reduces sensitivity to reward [19]. In contrast, CB1R stimulation elevates dopamine release via 2-arachidonyl-glycerol (2-AG) signalling, increasing motivation and reward-seeking behaviour [20].

In humans, some studies have found lower CB1R densities in the anterior cingulate cortex of MDD patients, in comparison to patients with other forms of psychopathology such as schizophrenia and bipolar disorder [21, 22]. In contrast, other postmortem investigations have observed enhanced CB1R densities in the prefrontal cortex [23, 24] and ventral striatum [25]. Furthermore, higher concentrations of 2-AG [24] have been found in the brains of suicide victims. Research has linked the activity of MAO-A and MAO-B enzymes to the ECS [26], and proposed targeting it for antidepressant therapy and identifying it as a biomarker for major depressive disorder [27–30]. One of the most direct evidence implicating the ECS in depression is the adverse effects of rimonabant, a CB1R antagonist used to treat obesity. Rimonabant use can worsen depressive symptoms, especially in those with a history of major depression [31]. Due to severe adverse effects on mood, including depression and suicidal thoughts, rimonabant was withdrawn from the market [32].

Several studies have identified a link between ECS and substance use disorders, particularly concerning positive reinforcement, relapse, and stress-induced craving [12, 33]. Brief exposure to alcohol has been shown to reduce endocannabinoid (eCB) signalling and lead to CB1R upregulation. However, prolonged alcohol exposure can trigger compensatory effects that enhance eCB signalling by increasing synthesis, reducing degradation, or both [34]. Additionally, alcohol exposure has been found to increase anandamide (AEA) formation [35], while oleoyl-ethanolamide (OEA) regulates physiological adaptations to alcohol exposure in animals [36]. Individuals suffering from AUD have lower CB1R availability than healthy controls, possibly due to receptor downregulation or increased CB1R occupancy by eCBs. Studies suggest that CB1R are persistently desensitized or reduced in AUD during abstinence [37].

Endocannabinoid modulation of brain regions involved in alcohol reward may be influenced by reduced fatty acid amide hydrolase (FAAH) activity, suggesting a role in hazardous alcohol use and AUD [38]. Preclinical studies have shown that FAAH activity and AEA levels regulate each other bidirectionally. Rats bred to prefer alcohol but not exposed to it showed decreased FAAH expression and activity and increased AEA levels [39]. Another study found that AEA inhibits FAAH via a lipoxygenase route, leading to reduced FAAH levels after elevated eCB release [40]. Furthermore, several studies indicated that the absence or partial deactivation of the FAAH gene leads to an increase in the consumption of ethanol, while also reducing the effects of ethanol intoxication and withdrawal symptoms [41–43]. Additionally, limited postmortem [25, 44] and clinical studies [45] suggest that reduced brain FAAH levels are associated with AUD. These findings suggest that inherited or acquired reductions in FAAH, as well as corresponding increases in endocannabinoids, may contribute to pathological drinking and could be used as a biomarker for AUD risk or severity.

Given that eCBs can travel through the blood-brain barrier and regulate the immune response in both the brain and periphery [46], it is reasonable to measure peripheral eCB concentrations to study how the ECS influences the development of MDD or AUD [47]. These concentrations can be easily and reliably measured in human serum and plasma, overcoming limitations in detecting brain eCB levels [48].

This systematic review aims to determine peripheral eCB levels in individuals with MDD and/or AUD. It also explores eCB compounds as diagnostic and prognostic biomarkers and potential therapeutic targets.

## Methods

### Search strategy

We conducted a systematic literature search from 1970 until October 22, 2023, starting from the earliest published manuscript in each database (MEDLINE’s earliest published manuscript dates back to August 19, 1970.). The following databases were consulted: MEDLINE, Web of Science and EMBASE. To conduct the search, we used specific terms related to the target population (“Major depressive disorder” and “Alcohol use disorder”) along with the chemical compounds (“Endocannabinoids”). These terms were combined using Boolean operators and then applied to each database without any date restrictions. The complete search strategy can be found in the Supplementary material.

The Preferred Reporting Items for Systematic Reviews and Meta-Analysis (PRISMA) guidelines served as guiding principles for reporting in our systematic review [49].

Two authors (JF and FF) conducted an initial screening of articles by reviewing their titles and abstracts. Full-text articles were obtained for all potentially relevant articles. In case of disagreement between the two authors, a third author (MT) was consulted to decide whether the full-text article should be obtained. Subsequently, the same two authors reviewed the full-text articles to determine their inclusion in the study. To ensure literature saturation, the electronic search was supplemented by a manual review of the reference lists from eligible publications.

### Eligibility criteria

Please note the following inclusion criteria for the study. Selected studies must involve human subjects who are adults aged 18 or older, with a minimum of 10 patients in the study. Participants must have a current diagnosis of Alcohol Use Disorder and/or Major Depressive Disorder, which must be diagnosed by a psychiatrist, or a structured clinical interview based on the Diagnostic and Statistical Manual of Mental Disorders (DSM) [50] or International Classification of Diseases (ICD) criteria. The studies must evaluate plasma or serum endocannabinoids. The acceptable types of study design include randomized and quasi-randomized trials, prospective or retrospective cohorts, longitudinal (one-arm) observational studies (time-series and before-after studies), and cross-sectional studies. Lastly, the manuscript should be written in English. The following studies are excluded: animal studies, studies in healthy volunteers, review papers, opinion pieces, comments, letters, editorials, conference abstracts, posters, case reports, and studies that do not report original data.

### Data extraction

The following details were gathered from the studies that were included: author names, publication year, study design, number and characteristics of patients, diagnostic method, any intervention performed during the study, the method used to measure serum/plasma endocannabinoid levels, and the outcome(s) related to MDD or AUD.

We carefully considered known variables that may affect endocannabinoid levels, including gender, age, race, BMI, and antidepressant use [51–53]. As the studies included different diagnostic groups, the outcomes varied depending on the psychiatric condition under study. In any case, peripheral eCB levels either from baseline or endpoint were extracted.

### Quality assessment

The Risk of Bias in Non-randomized Studies—of Interventions (ROBINS-I) tool was used to assess the risk of bias in included non-randomized trials [54]. The review process entailed six steps: (1) defining the research question by considering a target trial; (2) identifying the outcome and result being evaluated; (3) examining how confounders and co-interventions were handled for the specified result; (4) answering signalling questions for the seven bias domains; (5) making risk of bias judgments for each bias domain; and (6) giving an overall judgment on the risk of bias for the assessed outcome and result (categories include low, moderate, serious, critical risk of bias, or lack of information to make a judgment). For the included randomized studies, the revised Cochrane risk-of-bias tool for randomized trials (RoB 2) was used [55]. Similar to the ROBINS-I tool, the ROB 2 tool also followed six steps: (1) specifying the results being evaluated; (2) defining the effect of interest; (3) listing the information sources used for the assessment; (4) answering signalling questions for the five bias domains; (5) judging the risk of bias for each domain; and (6) evaluating the overall risk of bias for the result (categories include low risk, some concerns, or high risk of bias). The quality assessment was based on the primary efficacy outcome in the studies. The quality of observational studies that were eligible for inclusion was assessed using the Newcastle-Ottawa Scale (NOS) [56]. The studies were classified into three categories based on their NOS scores, which ranged from 0 to 9. Scores between 0 and 3 were considered low quality, scores between 4 and 6 were considered moderate quality, and scores between 7 and 9 were considered high quality. The scale assessed three key factors: selection of cohorts, comparability of cohorts, and outcome. This scale has also been adapted to evaluate the quality of cross-sectional studies [57], which were classified as low, fair, or good quality depending on their scores.

Due to the heterogeneous nature of the included studies, no meta-analysis was conducted. The protocol of the systematic review was registered with the International Prospective Register of Systematic Reviews (PROSPERO) under registration number CRD42023472381.

## Results

**Fig. 1:**
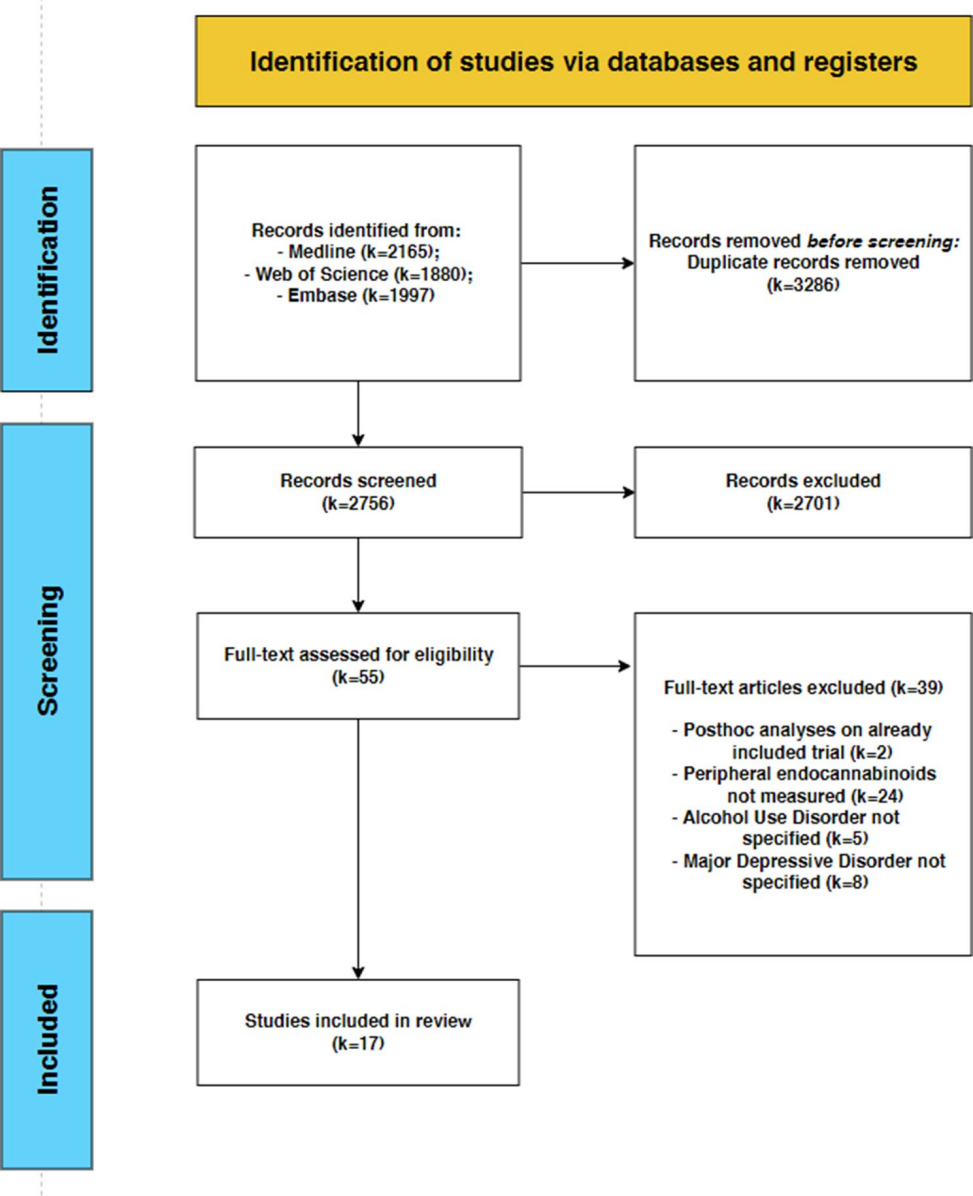
PRISMA flowchart of selected abstracts and articles

We retrieved a total of *k* = 2756 unique records through our systematic search in electronic databases. After screening titles and abstracts, *k* = 55 full-text articles were assessed for eligibility, and *k* = 17 articles were finally included in this systematic review. This process is described in the PRISMA flowchart (Fig. 1).

Twelve studies evaluated peripheral endocannabinoids in participants with MDD and five studies in participants with AUD. The detailed description of all studies included, and their main results can be found in Tables 1 and 2.

**Table 1:** Studies measuring peripheral endocannabinoids in major depressive disorder (MDD)

Study; (Country)	Hill et al., 2008 (Canada/ USA)	Hill et al., 2009 (Canada/USA)	Coccaro et al., 2018 (USA)	Romero- Sanchiz et al., 2019 (Spain)	Meyer et al., 2019 (USA)	Yang et al., 2019 (China)
Study design	Cross-sectional study	Cohort study	Cross-sectional study	Cross-sectional study	Open label, single-arm trial	RCT - double-blind non-placebo
Subjects characteristics	28 women diagnosed with depression (16 major depression)	15 women with major depressive episode (part of a larger project of immune response to acute stress)	115 participants with current or lifetime diagnoses of a psychiatric and/or personality disorder	69 patients with mild or moderate depression	17 women with self-reported Major Depressive Disorder	85 patients with diagnostic criteria of DSM-IV for Major Depressive Disorder
N: MDD / Control Age: mean (SD) Women: n (%) Caucasians: n (%) BMI: mean (SD)	16 / 28 27,6 years (9,7) 16 (100%) 6 (37,5%) 31,2 (8,1)	15 / 15 24,5 years (4,5) 15 (100%) 8 (53,3%) 26,9 (8)	22 / 60 35,7 (7,5) 60 (52,2%) 86 (74,9%) -	69 / 47 43,23 years (9,64) 49 (71%) 69 (100%) 25,27 (4,42	17 / - 40,8 years (14,8) 17 (100%) - 29.7 (8.0)	85 / - 40,94 years (14,95) 66 (77,6%) - 22,48 (3,52)
Control group	Women with no history of psychiatric illness matched on case-by-case basis with respect to age and ethnicity	Women with no history of psychiatric illness matched on case-by-case basis with respect to age and race	Healthy participants	47 healthy volunteers matched on case-by-case basis with respect age, gender and sex	No control	No control
Diagnostic Items	Depression Interview and Structured Hamilton by trained interviewers (DSM-IV)	Depression Interview and Structured Hamilton by trained interviewers (DSM-IV)	Structured Clinical Interview for DSM-5 Diagnoses (SCID-I) Clinical interview by a research psychiatrist	Structured Clinical Interview for DSM-IV Axis I Disorders (SCID-CV)	Self-reported MDD was confirmed by Mini-International Neuropsychiatric Interview (MINI).	Structured Mini- International Neuropsychiatric Interview (MINI) by an experienced psychiatrist
Endocannabinoids	AEA, 2-AG	AEA, 2-AG, PEA, OEA	AEA, 2-AG	OEA, POEA, AEA, DGLEA, DEA, DHEA, 2-AG, 2-LG, 2-OG	AEA, 2-AG, PEA, OEA, 2-OG	ALEA, EPEA, DHEA, LEA, AEA, 2-AG, 1-AG
Method used to quantify	(LC/MS) (serum)	(LC/MS) (serum)	(LC/MS/MS) (serum)	(LC/MS/MS) (plasma)	(LC/MS/MS) (serum)	LC-MS-MS (plasma)
Intervention	None	Trier Social Stress Test (TSST)	None	None	Exercise sessions performed in stationary bicycle (moderate- intensity exercise session or self- preferred intensity session).	3 groups taking different medications for 12 weeks: (1) 4 capsules of EPA (2) 4 capsules of DHA (3) 2 capsules of EPA and 2 of DHA
Main findings	- Decrease in serum 2-AG in women with major depression, significantly related to the duration of current depressive episode - No differences in serum AEA between groups. - Serum AEA exhibited highly significant negative correlation with scores on both Hamilton variable for cognitive anxiety and somatic anxiety	- Serum concentrations of both 2-AG and AEA were significantly reduced in depressed women relative to controls. - Stress exposure significant increase in circulating 2-AG concentration in women immediately following administration of TSST, but not 30 min after stress cessation. - PEA and OEA significant decrease during stress recovery phase (30 min after stress cessation)	- Modest, statistically significant, relationship between composite affect regulation scores and both AEA and 2-AG. - No significant difference in AEA, nor 2-AG in composite State depression, nor anxiety score. - No significant difference in circulating levels of AEA, nor 2-AG, between healthy and psychiatric group.	- Plasma OEA concentrations were found to be elevated in depressed patients and to correlate with somatic symptoms of depression. - Plasma content of DGLEA and 2- AG were significantly elevated in depressed patients. - The elevation observed in plasma concentrations of both OEA and 2AG was associated with ISRS at the time of recruitment	- Moderate exercise resulted in significant increases in circulating levels of AEA and OEA in women with MDD. - Elevation of AEA in moderate exercise session related to decreases in feelings of depression, confusion, fatigue, total mood disturbance and state anxiety. - Elevation in 2-AG were also significantly associated with reduction in feelings of depressed mood, confusion and total mood disturbance up to 30 min post moderate exercise session.	- Clinical remission was significantly higher in the EPA and EPA + DHA groups than the DHA group. - EPA and EPA + DHA treatments increased EPEA levels compared to DHA treatment while EPA + DHA treatment increased the DHEA levels more than EPA treatment. - Plasma EPEA levels were positively correlated with rates of clinical remission. - Comparing to the baseline, post-treatment plasma AEA levels were decreased in EPA, DHA and EPA + DHA groups.
**Study; (Country)**	**Harfmann et al., 2020 (USA)**	**Zajkowska et al., 2020 (UK)**	**Kang et al., 2021 (USA)**	**Bersani et al., 2021 (Italy)**	**Lazary et al., 2021 (Hungary)**	**Behnke et al., 2022 (Germany)**
Study design	Cross-sectional study	Cohort study	Cohort study	Cohort study	Open label, single-arm trial	Cross-sectional study
Subjects characteristics	44 grief participants (within 13 months following the death of a loved one)	70 patients with chronic HCV infection and compensated liver disease under interferon-alpha treatment	44 grief participants (within 13 months following the death of a loved one) aged 50 years and older.	12 participants diagnosed with Major Depressive Disorder (DSM-V criteria)	18 adult subjects diagnosed with treatment-resistant major depression	20 women diagnosed with major depressive disorder
N: MDD / Control Age: mean (SD) Women: n (%) Caucasians: n (%) BMI: mean (SD)	21 / 17 65,8 years (9,2) 17 (39%) 41 (93%) 29,3 (4,8)	28 / 41 43,77 years (1,49) 17 (24,3%) - -	21 / 20 66,40 years (8,8) 30 (68,2%) 41 (93,2%) -	12 / 12 58,67 years (12,12) 3 (25%) - 22,58 (4,58)	18 / - 47,7 years (12,1) 13 (72,22%) - 23,3 (4,5)	20 / 24 33 years (26,5) 20 (100%) - 25,3 (6)
Control group	17 healthy controls (no lifetime history of psychiatric illnesses)	41 healthy controls matched for age and gender	20 healthty controls	12 healthy controls (age and sex matched)	No control	24 healthy controls
Diagnostic Items	Structured Clinical Interview for DSM-5 Research Version	Mini International Neuropsychiatric Interview (MINI) Major Depression section	Structured Clinical Interview for DSM-5 Research Version	Italian version of the Mini International Neuropsychiatric Interview (MINI) DSM-V	DSM- IV criteria and determined by experienced psychiatrists	German Structured Clinical Interview; Translation of the English SCID-5-CV (not validated)
Endocannabinoids	AEA, 2-AG	AEA, 2-AG	AEA, 2-AG	AEA, 2-AG	AEA, 2-AG	AEA, 2-AG, PEA, SEA, OEA
Method used to quantify	(LC/MS/MS) (serum)	(LC/MS) (serum)	(LC/MS/MS) (serum)	(LC/MS/MS) (plasma)	(LC/MS/MS) (serum)	(LC-MS/MS) (plasma)
Intervention	None	- Weekly, subcutaneous injection of interferon-alpha (1.5 mg/Kg) - Daily Ribavirin, administered orally in doses ranging from 800 to 1400 mg	None	Escitalopram was prescribed to all patients at the dose of 10 mg/ day at first visit	- Repetitive transcranial magnetic stimulation (rTMS) treatment five days a week during a total of ten sessions. - 18 treated with antidepressive treatment (100%)	None
Main findings	- Serum AEA significantly elevated in grief participants compared to healthy controls. - AEA concentrations positively associated with HAM-D and HAM-A scores in a significant way in grief group. - No significant differences in 2-AG serum levels between groups and no associations with clinical measures.	- AEA and 2-AG increased significantly during treatment, and the pattern of change was different - No significant difference in AEA and 2-AG levels between patients with and without interferon-alfa induced depression before, during, and after treatment.	- Serum AEA concentrations were significantly increased in the Grief-High-Loneliness group compared with healthy controls. - Grief participants revealed a positive association between loneliness scores and serum AEA concentrations - The grief participants with high loneliness at baseline and high serum 2-AG concentrations had a greater rate of improvement in ICG scores over 26 weeks.	- No significant differences in basal serum plasma of AEA/2-AG between depressive and controls. - Plasma levels of 2-AG and AEA did not change significantly overtime in response to escitalopram treatment. - 2-AG showed a significant negative correlation with BDI total scores at basal point. - No correlation between AEA levels and BDI scores.	- Association between changes in 2-AG level and reduction of depressive and anxious symptoms immediately following completion of a 2- week rTMS treatment showed a strong trend. - A greater increase in 2-AG concentrations corresponded to a greater decrease of symptoms. - Strongest association between change of 2-AG and improvement of anxiety	- Women with MDD had higher levels of circulating AEA than non-depressed women, while the groups did not significantly differ in the levels of 2-AG, PEA, SEA, and OEA. - Circulating AEA concentrations were higher in women showing more depressive symptoms (BDI)

**Table 2:** Studies measuring peripheral endocannabinoids in Alcohol Use Disorder (AUD).

Study; (Country)	Mangieri et al., 2009 (USA)	Spagnolo et al., 2016 (USA)	Garcia-Marchena et al., 2017 (Spain)	Brellenthin et al., 2019 (USA)	Best et al. 2020., (Canada)
Study design	Cohort study	Cohort study	Cross-sectional study	Randomized clinical trial	Cohort study
Subjects characteristics	Treatment-engaged patients with current alcohol dependence, recently abstinent.	AUD + post-traumatic stress disorder (FAAH genotype: C385 homozygotes (CC)) and following detoxification	Abstinent (at least 4 weeks) alcohol-dependent patients under current treatment intervention.	Substance use disorder under exercise protocol.	Alcohol use disorder participants
N: AUD / Control Age: mean (SD) Women: n (%) Caucasians: n (%) BMI: mean (SD)	12 / 11 39,17 years (6,9) 2 (17%) 9 (75%) 26,43 (4,59)	24 / 25 39,5 years (7,9) 11 (45,8%) 12 (50%) -	79 / 79 49,13 years (9,6) 27 (34,2%) 79 (100%) 25,83 (4)	11 / 10 35,1 years (10,2) 5 (45,5%) 7 (63,6%) 30,2 (5,9)	14 / 25 46,93 years (10,87) 1 (7,1%) 13 (92,9%) 27,66 (5,71)
Control group	Healthy social drinkers (up to six drinks weekly).	AUD + post-traumatic stress disorder (FAAH genotype: 385 A carriers (AX)) and following detoxification	Healthy volunteers with no history of drugs abuse.	Substance use disorders treated as usual (not exercise protocol).	Healthy participants.
Diagnostic Items	Structured Clinical Interview (SCID).	Structured Clinical Interview (SCID).	PRISM (Psychiatric Research Interview for Substance and Mental Disease, spanish version).	DSM-IV criteria for substance use disorder.	DSM-IV criteria for alcohol use disorder.
Endocannabinoids	AEA, OEA, 2-AG	AEA, 2-AG, PEA, OEA.	PEA, SEA, OEA, POEA, AEA, LEA, DHEA, DGLEA, DEA	AEA, 2-AG	AEA, OEA, DHEA
Method used to quantify	LC-MS-MS (plasma)	LC/MS/MS (serum)	LC-MS-MS (plasma)	(LC/MS/MS) (plasma)	(LC/MS/MS) (plasma)
Intervention	Exposure to guided imagery scripts for alcohol cues, personal stressors and neutral relaxing states (Scene Construction Questionaire).	Exposure to auditory guided imagery script challenge sessions, using personalized stress-, alcohol-associated or neutral stimuli.	None	Exercise protocol: 18 sessions at the same time of day during 3 weeks: Incline walking performed on a private treadmill in the laboratory.	None
Main findings	- Baseline plasma AEA markedly reduced in abstinent alcoholics compared to control group. - In healthy drinkers, alcohol cue-induced craving was accompanied by a marked elevation in circulating levels of AEA - No imagery-induced AEA mobilization observed in patients with alcohol dependence.	- Robust main effect of genotype on AEA levels, with 385 A carriers showing increased serum AEA, OEA and PEA levels throughout the course of the procedure. - Anxiety response declined more rapidly in 385 A carriers. - Subjects carrying the low-expressing 385 A variant exhibited decreased arousal compared to C homozygotes (PSSI scores).	- Abstinent alcohol-dependent patients had significant higuer plasma concentrations of all acyl ethanolamines than control subjects (only OEA, AEA and DEA were explanatory variables). - OEA, AEA and DEA concentrations were negatively correlated to the duration of alcohol abstinence.	- AEA levels significantly increased acutely after exercise, but not quiet rest. - There were not acute changes in 2-AG.	- Brain FAAH activity was significantly lower in AUD participants, and correlated negatively with number of standard drinks per week. - Significantly higher plasma concentrations of AEA, OEA and DHEA in early abstinence compared to healthy controls and negatively correlated with brain FAAH activity.

*Abbreviations: HCV, hepatitis C virus; DSM-V, diagnostic and statistical manual of mental disorders; SCID, Structured Clinical Interview for DSM Disorders; HAM-D, Hamilton Depression Rating Scale; BDI, Beck Depression Inventory; ICG, Inventory of Complicated Grief; MDD, major depressive disorder; AUD, Alcohol Use Disorder BMI, body mass index; SD, standard deviation; LC/MS, liquid chromatography-mass spectrometry; LC/MS/MS, liquid chromatography with tandem mass spectrometry; AEA, anandamide; 2-AG, 2-arachidonyl-glycerol; OEA, oleoyl-ethanolamide; FAAH, fatty acid amide hydrolase; EPEA, eicosapentaenoylethanolamide; PEA, palmitoylethanolamide; DGLEA, dihomo-gamma-linolenoyl ethanolamide; DEA, docosatetraenoyl ethanolamide; DHEA, docosahexaenoyl ethanolamine; POEA, palmitoleoyl ethanolamide

### AUD and MDD, gender differences

Peripheral endocannabinoids were measured in a total of 170 AUD patients and 359 MDD patients. Notably, there is no scientific literature reporting peripheral endocannabinoid levels in patients with comorbid major depressive disorder and alcohol use disorder. Moreover, excluding participants with current or past alcohol abuse was common practice in studies of major depressive patients, except for one study that excluded only severe substance use disorders [58]. On the other hand, two studies in AUD participants reported 35 lifetime mood disorders [59, 60], but did not provide information on their status or complete definition.

Gender was not reported for 16 AUD and 92 MDD patients. Among the remaining 154 AUD participants, 51.3% (79) were women, while among the remaining 267 MDD patients, 79% (211) were women. Out of all the studies that were included, only three showed differences in eCBs based on gender. Romero-Sanchiz and colleagues [58] found that the concentration of docosahexaenoyl ethanolamine (DHEA) was significantly higher in men than in women. Moreover, García-Marchena et al. [59]. , observed a significant main effect of sex factor on palmitoleoylethanolamide (POEA) concentration with higher concentration in women relative to men. Finally, in a study by Best and his team [45], gender-based differences revealed a trend for higher FAAH levels in women, but this did not have a significant effect overall.

### Antidepressant treatment

Antidepressant treatment was reported in a total of 135 patients (96 MDD and 39 AUD patients). Two studies [61, 62] did not report antidepressant use, and six studies [45, 60, 63–66] excluded patients taking antidepressants. In two other studies [67, 68], all MDD patients were being treated with antidepressants.

During their research, Romero-Sanchiz’s team [58] discovered a link between the use of SSRIs and higher levels of OEA, 2AG and dihomo gamma-linolenoyl ethanolamide (DGLEA) in the plasma during the recruitment process. However, in a separate study, Bersani et al. [68]. noted no significant variations in endocannabinoid plasma levels with escitalopram treatment over time. Meyer et al. [69]. described changes in endocannabinoids throughout the exercise sessions based on the use of antidepressants but did not report any statistical differences. While some studies attempted to specify the type of antidepressant used [70], most did not analyse this concerning peripheral eCB levels.

### 2-AG

#### Major depressive disorder

Some studies have yielded conflicting results regarding the levels of peripheral 2-AG in patients with MDD when compared to healthy individuals. Hill et al. [65, 66] observed a noteworthy reduction in 2-AG levels in all-female MDD patients. However, Romero-Sanchiz et al. [58]. reported significantly higher 2-AG levels in MDD patients, contradicting Hill et al.‘s findings. Different cohort compositions and therapy access in previous clinical studies can account for varying profiles of plasma 2-AG concentrations. However, most studies did not show significant differences between MDD individuals and healthy controls [61, 63, 68, 71]. Coccaro et al. [61], did not compare eCB levels in depressed individuals and healthy controls, thus data analysis was not possible.

#### Severity and chronicity of depressive symptoms

In a study conducted by Bersani and his team [68], they discovered a significant inverse relationship between the initial levels of 2-AG and self-reported depressive symptoms, measured by Beck Depression Inventory (BDI) scores. Additionally, Kang et al. [70] reported that individuals with high levels of 2-AG experienced a faster reduction in grief symptoms over 26 weeks if they reported higher levels of loneliness at the beginning of the study. Furthermore, Meyer et al. [69] found that higher 2-AG levels were associated with lower depressed mood, confusion, and total mood disturbance for up to 30 min after moderate exercise sessions.

Surprisingly, Hill et al. [65] showed that female patients with MDD episodes of mild to moderate severity showed higher levels of AEA but not 2-AG when compared to non-depressed controls. Partially resembling these findings, Behnke et al. [71], found higher AEA levels and a trend for higher OEA levels but no alteration in 2-AG in women with MDD episodes of mainly mild to moderate severity. Hill et al. [65] also found that 2-AG levels decrease as major depressive episodes progress chronically, but not in cases of minor depression. Reduced activity in the ECS system may lead to less stress buffering and more persistent depressive symptoms. Thus, concentrations of circulating 2-AG may be a particular feature of depression severity and chronicity.

#### Stress and inducibility of 2-AG

Hill et al. (2009) [66] showed that stress exposure led to a significant increase in 2-AG concentration in women, depressed or not, immediately after Triel Social Stress Test (TSST) administration. However, this increase was not observed after 30 min. The diagnosis of depression did not impact endocannabinoid content in response to stress.

Lazary et al. [67]. reported that 10-day Repetitive Transcranial Magnetic Stimulation (rTMS) treatment increased serum 2-AG levels in 18 patients with treatment-resistant depression. Higher 2-AG levels were associated with reduced symptoms of depression, anhedonia, neurocognitive, and anxiety, with the strongest link being anxiety symptoms. The study suggests that it is the endocannabinoid system’s inducibility and not the initial serum content that is associated with rTMS treatment’s antidepressant effect.

#### Alcohol use disorder

Included studies found no significant correlation between AUD diagnosis, AUD severity or length of abstinence and peripheral levels of 2-AG [60, 62, 72].

### AEA

#### Major depressive disorder

Studies have shown inconsistent results for AEA peripheral levels in patients with MDD compared to controls. A study conducted by Hill et al. in 2009 [66] found that the basal serum concentration of AEA in 15 women with major depressive disorder was significantly lower compared to that of healthy controls. However, a more recent study by Behnke et al. in 2023 [71] reported higher circulating AEA levels in 20 women with MDD compared to non-depressed women. On the other hand, most studies [58, 61, 63, 65, 68] showed no significant difference in MDD diagnosis compared to controls.

#### Anxiety symptoms

Included studies suggest that anxiety symptoms are negatively correlated with AEA levels in the peripheral system of humans. Hill and colleagues [65] discovered that there is a negative connection between serum AEA and anxiety symptoms in 28 depressed women who have not undergone treatment. According to the Hamilton Depression Rating Scale (HDRS), those with higher levels of anxiety showed lower serum AEA content for both cognitive and somatic anxiety. Besides, Meyer and colleagues [69] found a significant increase in AEA following moderate-intensity exercise, which was associated with decreases in anxiety. Moreover, a genotype study [62] of two cohorts, consisting of 25 low-expressing FAAH variant (385 A carriers) and 24 common FAAH variant, showed that 385 A carriers had higher serum AEA levels throughout the study. Although both groups initially had similar anxiety levels, 385 A carriers experienced a faster decline in anxiety. However, Harfmann et al. [73]. found increased serum AEA levels in the blood of individuals with grief, along with a positive correlation with anxiety scores. The authors suggested this may be a protective mechanism against negative stress responses.

#### Depression severity

Studies analysing AEA levels in relation to depression severity do not consistently yield results. In 2019, a study by Romero-Sanchiz et al. [58]. found that AEA levels were higher in moderate depression patients than those with mild depression and associated with severe somatic symptoms. Kang et al. [70] found a positive correlation between loneliness scores and serum AEA concentrations in grievers, but this association ceased to be significant after adjusting for depression severity. Similarly, Harfmann et al. [73] showed that AEA concentrations were positively associated with HDRS depression scores in a significant way in the grief group.

However, Hill et al. [65] found that patients with minor depressive disorder had significantly increased serum levels of AEA. In another study by Meyer et al. [69], an increase in AEA was broadly associated with a decrease in feelings of depression, fatigue, and overall mood disturbance resulting from exercise in depressed women. Similarly, in a clinical trial conducted by Yang et al. [64], AEA levels were decreased after 12 weeks of eicosapentaenoic acid (EPA) and docosahexaenoic acid (DHA) treatments. The three groups showed a significant overall effect on the cumulative remission rate as measured by HDRS depression scores.

#### Alcohol use disorder and length of abstinence

Research has shown that AEA significantly increases during early abstinence in AUD compared to healthy controls. However, reliability decreases for longer AUD abstinence periods.

According to Garcia-Marchena [59], a study of 79 abstinent (4 weeks at least) alcohol-dependent patients found that they had significantly higher plasma concentrations of AEA compared to control subjects. AEA concentrations were negatively correlated with the duration of alcohol abstinence. In another study, Best and colleagues [45] reported that 14 individuals in early abstinence (with a 5-day mean) had significantly higher plasma concentrations of AEA compared to 25 healthy controls. There were no significant differences in AEA plasma levels between individuals with AUD and healthy controls during longer abstinence (2–4 weeks). Furthermore, an older study by Mangieri and colleagues [60] found baseline plasma AEA significantly reduced in 12 abstinent (4 weeks) alcoholics compared to 11 healthy social drinkers. Other studies [62, 72] did not compare AUD diagnosis and AEA levels with those of healthy control, and length of abstinence was not shown.

The studies included in the analysis revealed a significant negative correlation between peripheral levels of AEA and FAAH activity. Best et al. [45] found that AEA concentrations were negatively correlated with brain FAAH activity in individuals with AUD during early abstinence, but not during longer abstinence. Similarly, in the genotype study conducted by Spagnolo et al. [62], participants with the 385 A FAAH variant had higher serum AEA levels during the procedure. Interestingly, in the study conducted by Mangieri and colleagues [60], alcohol cue-induced craving was accompanied by a marked elevation in circulating levels of AEA in healthy drinkers, but not in alcohol-dependent patients.

### OEA

#### Major depressive disorder

Four studies have investigated the peripheral levels of OEA in people with MDD, and they have produced conflicting results. One study [58] found that depressed patients had higher plasma concentrations of OEA, which were linked to more severe depression and somatic symptoms. However, two other studies [66, 71] did not find any significant difference in OEA levels between patients with MDD and healthy controls. Another study [69] showed that moderate-intensity exercise led to an increase in the circulating OEA in women with major depressive disorder, but this increase was not strongly correlated with clinical improvements.

#### Alcohol use disorder and FAAH activity

Four studies have analysed OEA levels in patients with AUD, and the evidence they provide suggests a correlation between increased OEA levels in AUD patients and decreased FAAH activity. Garcia-Marchena [58] reported that abstinent alcohol-dependent patients had significantly higher plasma concentrations of OEA than control subjects, and OEA levels were negatively correlated to the duration of alcohol abstinence. No effects of psychiatric comorbidity were related in OEA concentrations, but major depressive disorder lacks a complete definition, and comparing results is not feasible. On the other hand, Mangieri et al. [60], did not report changes in OEA levels in AUD compared to healthy controls.

Best and colleagues [45] showed that during early abstinence from alcohol dependence, plasma levels of OEA were found to be higher when compared to healthy controls. This increase in OEA levels was negatively correlated with brain FAAH activity, which was similar to AEA. However, there was no significant difference in OEA levels between long-term abstinent individuals and healthy controls. Spagnolo also reported increased OEA levels in patients with AUD who had low-expressing FAAH variant [62].

### Eicosapentaenoylethanolamide (EPEA)

In a randomized controlled trial conducted by Yang and colleagues [64], EPEA was measured in the plasma of 88 participants with major depression who were given DHA, EPA or a combination of both. The study found that EPEA levels were increased in all treatment groups, with the EPA-containing treatments showing the highest increase. The study also found a positive correlation between EPEA levels and clinical remission rates, suggesting that EPEA could be a potential endogenous therapeutic target for treating major depressive disorder. Thus far, no other studies have examined the peripheral levels of EPEA in patients with MDD or AUD.

### Other eCBs and endocannabinoid-like compounds

#### Palmitoylethanolamide (PEA)

Three studies conducted on individuals with MDD failed to yield significant results with regards to the levels of peripheral PEA observed. PEA levels were similar in depressed and non-depressed women in two studies [66, 71]. Meyer et al. [69] found no changes in PEA levels after exercise. During recovery from stress in depressed women, Hill et al. [66] found a significant reduction in PEA levels, which was similar to OEA.

One study in AUD patients found a direct correlation between PEA levels and AUD [58], while another study found an inverse correlation between PEA levels and FAAH activity, like the cases of AEA and OEA [62].

#### Dihomo-gamma-linolenoyl ethanolamide (DGLEA)

In one study [58], it was observed that depressed patients had significantly higher levels of DGLEA in their blood compared to the control group. The study also found that patients who were taking antidepressants had higher levels of DGLEA compared to those who were not receiving antidepressant therapy. In another study [58], it was found that abstinent alcohol-dependent patients had significantly higher levels of all DGLEA in their plasma than the control group. No other studies have been conducted on the peripheral levels of DGLEA in MDD or AUD patients.

#### Docosatetraenoyl ethanolamide (DEA)

Only two studies examined peripheral DEA levels. One found higher plasma DEA levels in abstinent alcohol-dependent patients compared to controls, negatively correlated with abstinence length [59]. No significant differences in peripheral DEA levels were observed between MDD participants and healthy controls [58].

#### Docosahexaenoyl ethanolamine (DHEA)

Research showed that early abstainers from alcohol displayed high plasma concentrations of DHEA, which negatively correlated with brain FAAH activity [45]. However, there was no significant difference in DHEA levels between longer abstainers and healthy controls with AUD.

Peripheral DHEA levels were found to be similar in MDD individuals and healthy subjects [58], and treatment with EPA and DHA did not lead to clinical remission rates despite increasing DHEA levels [64].

#### Palmitoleoyl ethanolamide (POEA)

In the only study that measured POEA in MDD patients, it was found that the severity of depression was positively correlated with POEA levels [58]. Additionally, in the only selected study that measured POEA in AUD patients, it was observed that POEA levels were significantly higher in AUD patients compared to healthy controls [59].

The rest of the analysed chemical compounds did not reach any significant result to our systematic review.

### Quality assessment

Several clinical studies have been evaluated for their quality using various tools. The randomized clinical trials conducted by Yang et al. [64] and Brellenthin et al. [72], were found to have some concerns and moderate risk of bias, respectively, according to the ROB-2 tool. The non-randomized clinical trials conducted by Meyer et al. [69] and Lazary et al. [67], were rated with serious and moderate risk of bias, respectively. The quality assessment of cohort studies was conducted using the NOS tool, which revealed three studies [62, 66, 70] with good quality, three studies [45, 63, 68] with fair quality and one study [60] with poor quality. Additionally, cross-sectional studies were evaluated using the NOS tool, which revealed two studies [58, 59] with fair quality and four studies [61, 65, 71, 73] with poor quality.

For a comprehensive understanding of quality assessment, please refer to the Supplementary material (Tables 1, 2, 3 and 4).

## Discussion

Studies on patients with major depressive disorder (MDD) or alcohol use disorder (AUD) have found dysregulation in peripheral levels of endocannabinoid (eCB) and endocannabinoid-like compounds. These dysregulations may be influenced by various factors such as gender, chronicity, symptom severity, comorbid psychiatric symptoms, length of abstinence in the case of AUD, and stress-inducibility.

### Major depressive disorder

Our systematic review found conflicting results regarding peripheral eCBs in patients with MDD compared to healthy controls. It should be noted that preclinical studies typically associate changes in the ECS with melancholic depression, while the diagnostic criteria for MDD include various subtypes of clinical phenotypes [74]. The combination of data from all depressed individuals involved in the review may have obscured a more accurate connection between MDD and peripheral eCBs.

As mentioned by Zajkowska et al. [63], studies reporting eCB deficiency in depression did not investigate inflammation-induced depression. As previous studies have shown [75], increased inflammation can lead to elevated eCB levels and elevated inflammation has been reported in a subgroup of depressed patients who are not responsive to antidepressant treatment [76]. Alcohol-induced depression may be a specific type of depression that is caused by dysregulation of the endocannabinoid system, but scientific data is lacking. Opportunely, diagnostic tools such as the Psychiatric Research Interview for Substance and Mental Diseases (PRISM) [77] have been developed to diagnose alcohol-induced depression.

A study conducted by Pavón et al. [78] was not included in this review because the authors did not provide a clear definition of MDD. However, they used PRISM tool for assessing primary and cocaine-induced mood disorders. The study found that significant increases in OEA and POEA were only observed in individuals with cocaine-induced mood disorders as compared to those without mood disorders. This indicates that the increased levels of eCBs in individuals with cocaine use disorder were strongly potentiated by mood disorders, especially those induced by cocaine. There is a lack of scientific literature on peripheral eCB levels in patients with comorbid MDD and AUD. This gap in information hinders our understanding of the potential role of eCBs in treating these conditions.

### Depressive symptoms

Some selected studies suggested an inverse relationship between peripheral 2-AG levels and the severity of depressive symptoms [68–70], as well as longer depressive episodes [65]. However, a recent study conducted by Fitzgerald et al. [79] has found that individuals who experience trauma and have higher peripheral levels of 2-AG are more likely to suffer from depression six months later. Interestingly, there was no observed relationship between concurrent measures of circulating eCBs and depression after six months. This finding contrasts with prior studies which found that individuals with established, chronic depression had diminished circulating 2-AG levels [65, 66].

Selected studies showed conflicting results on the link between AEA and OEA levels and depressive symptoms. Other studies in healthy individuals [80] or with fibromyalgia [81] have found that high levels of circulating AEA are positively linked to depressive symptoms.

To better understand these biomolecules and their association with MDD, further research is needed to explore possible non-linear associations between ECS regulation and MDD severity, covering different phases of depressive disorders. The ECS has a unique feature called retrograde signalling where signalling starts from postsynaptic neurons and affects presynaptic terminals. AEA and 2-AG are produced in postsynaptic neurons and released into the synaptic space. They then travel in a retrograde direction to the presynaptic terminal and interact with CB1R, leading to a decrease in neurotransmitter release [82]. Retrograde signalling is used to synthesize these lipids as needed, and peripheral levels could be affected by physical or psychological stressors [83].

### Antidepressants

Several studies in our systematic review demonstrated peripheral eCBs changes related to antidepressant therapy [58, 64, 67]. Romero-Sanchiz and colleagues [58] reported that the increase in 2-AG and OEA levels was significant because these lipids have shown antidepressant activity in preclinical models of affective disorders [84].

Yang et al. (2019) [64] have identified EPEA as a promising endogenous target, paving the way for research in this field. EPEA could contribute to the therapeutic effects of Omega-3 polyunsaturated fatty acids (ω–3 PUFAs). This finding supports previous clinical [85] and preclinical studies [86], which demonstrated a remarkable increase in the formation of DHEA and EPEA in blood [87] after administering ω–3 PUFAs. Yang et al. suggested that ω–3 PUFAs have antidepressant effects by regulating endocannabinoid levels, as purported in preclinical studies [88, 89]. EPEA or DHEA may bind to CB1R, which can have an anti-inflammatory or immune-modulating effect, being more active than PUFA precursors [90]. ECBs may increase monoaminergic neurotransmission and accumulate in the brain, enhancing the reuptake of serotonin, norepinephrine, and dopamine [88]. Therefore, increased peripheral EPEA levels may be a potential target for treating depression, pending further research.

In 2018, Ghazizadeh-Hashemi et al. [91] published the results of a 6-week, double-blinded, placebo-controlled RCT that investigated the effect of PEA as an add-on treatment for 54 MDD patients. All patients received up to 40 mg citalopram per day, and half of them also received 600 mg of PEA twice daily. The study showed that the PEA group experienced a significantly larger improvement in depressive symptoms compared to the placebo group, although there was no difference in the number of remissions between the two treatments. The study did not measure eCB levels.

Directing attention to the ECS may lead to a promising treatment of depression. However, it is important to note that the studies selected for analysis did not provide a consistent definition of antidepressant treatment, making it imperative for future studies to establish a clear definition to ensure accurate and reliable results.

### Alcohol use disorder

Based on the data from the included studies, the ECS may have a significant role in the development of alcohol use disorder [59]. However, the involvement of eCBs is complicated by their effect on the modulation of stress-induced alcohol craving [60], length of abstinence [45], and FAAH activity [62].

The study conducted by García-Marchena et al. [59]. has suggested that alcohol consumption affects the biosynthesis or degradation pathways of all eCBs. Meanwhile, in other selected studies [45, 62], it has been observed that FAAH activity plays a crucial role in regulating the peripheral levels of its substrates. It is unclear whether chronic alcohol use initially elevates peripheral endocannabinoids through increased biosynthesis [92], mobilization in peripheral tissues [93], or by reducing FAAH activity and/or gene expression [35]. Low FAAH levels in AUD may result from changes in endocannabinoids as a compensatory response to decreased CB1R stimulation. This may increase endocannabinoid tone and restore CB1R activity.

In the study conducted by Best and colleagues in 2020 [45], the use of PET imaging with the FAAH radiotracer [11 C]CURB revealed that individuals who had lower levels of FAAH in their brain and higher levels of AEA in circulation were more likely to consume larger amounts of alcohol. These findings support preclinical studies suggesting endocannabinoid involvement in alcohol-seeking behaviours [38, 94]. Decreased endocannabinoid metabolism may promote increased drinking or reflect an adaptation to alcohol consumption.

The investigation of altered endocannabinoid signalling is crucial in understanding the perpetuation of alcohol use disorder in humans. Those with the FAAH C385A polymorphism, which reduces FAAH function, are at an increased risk for AUD due to higher alcohol intake and dependence severity [95]. Some clinical studies in youth have linked the FAAH minor allele variant to increased consumption of alcohol and other drugs [96, 97]. Furthermore, greater risks for binge drinking, drinking initiation, and escalation were associated with slow FAAH activity in another study [98]. Crosstalk between the dopaminergic and endocannabinoid systems has been linked to alcohol response, with FAAH polymorphism altering D3 receptor levels in humans and rodents [99]. It is essential to explore potential endocannabinoid-mediated pathways that contribute to the risk of developing alcohol use disorders in future research.

OEA has therapeutic potential in treating negative effects of alcohol abuse, including cognitive decline, neuroinflammation, withdrawal responses, motivation, and relapse [100]. Similarly, CB1R antagonism decreases voluntary intake of alcohol in rodents and suppresses dopamine release [101]. The potential for treating SUDs with neutral CB1R antagonists, CB2R agonists, and nonselective phytocannabinoids has been demonstrated in experimental animals. Accumulating evidence supports their therapeutic effectiveness and justifies their exploration as viable treatment options [102].

### Anxiety

Some included studies [62, 65, 69] have found an inverse relationship between anxiety symptoms and peripheral AEA content in humans. Based on preclinical research, increased AEA signalling in the brain reduces anxiety and improves mood [103–105]. This suggests that higher levels of AEA in the bloodstream may have similar effects.

Several studies have found that individuals with anxiety have lower peripheral AEA content, and those with PTSD and lower AEA content have more severe symptoms [69, 106, 107]. Exercise-induced increases in AEA concentrations are linked to positive affect in healthy individuals [108, 109]. Interestingly, individuals with PTSD fail to exhibit exercise-induced increases in circulating 2-AG concentrations, while elevations in AEA are still preserved [110].

Harfmann et al. [73]. proposed that higher serum AEA levels indicate an active ECS response in people experiencing grief. AEA signalling may help transition to integrated grief, and a positive correlation between serum AEA levels and depressive/anxiety symptoms was observed only in those with low grief symptoms.

The signalling ability of AEA to reduce anxiety has been observed to be highly specific to the stressful nature of the environment. This implies that blocking FAAH using either pharmacological or genetic methods can be more effective in reducing anxiety-related behaviours when dealing with challenging environmental conditions or after experiencing overt stressors [111, 112]. Elevating AEA signalling has been shown to effectively reduce anxiety caused by both acute and chronic stress [113, 114], and AEA may have an inverse relationship with the severity of anxiety experienced [115]. These findings emphasize the significance of AEA levels in evaluating anxiety and related disorders.

### Inducibility of endocannabinoids

Our systematic review revealed that the ECS could be induced by physical [69, 72] or psychological stress(Hill, Miller, et al., 2009; Mangieri et al., 2009; Spagnolo et al., 2016), and rTMS treatment [67]. A growing body of evidence suggests a significant interplay between physical exercise and the ECS in both central and peripheral systems. Physical exercise-induced activity in the ECS is crucial in regulating motor activity, nociception, and emotional processing [116].

Stress triggers an increase in peripheral 2-AG levels, [66, 117, 118], and enhanced CB1R signalling moderates the emotion regulation brain circuit, resulting in faster termination of stress responses(deRoon-Cassini et al., 2020). One study in healthy humans [107] found that psychological stress increased circulating levels of 2-AG and AEA. Additionally, Hill et al. [66] reported that 2-AG concentrations were significantly elevated in both depressed and non-depressed women after stress exposure. Moreover, in healthy individuals, the increase in 2-AG - but not AEA - following acute exercise was negatively correlated with depressed mood [119]. After experiencing trauma, Fitzgerald et al. [79] found that ECS is significantly activated via 2-AG, and down-regulation of CB1R signalling could increase the risk of developing long-term depression. Other studies have reported AEA -but not 2-AG- increases after physical [69, 72] and psychological stress [60]. According to Brellenthin et al. [72], the lack of an acute 2-AG response to exercise in SUD patients might indicate dysfunction in the ECS and contribute to aberrant acute stress responses. This idea is supported by Crombie et al. [110], who found that levels of 2-AG increased after exercise in healthy individuals, but not in PTSD patients.

Upon exposure to stress, there is evidence of co-regulation between endocannabinoids and other biomolecules, whose activation occurs in a specific sequence [120]. In the study by Lazary et al. [67], serum AEA levels decreased temporarily after rTMS treatment but returned to pre-treatment levels after 2 weeks. Although there was no significant change in 2-AG concentration, increased 2-AG at 2 weeks was significantly linked to symptom improvement. The mechanism described in this context plays a crucial role in the stress response. When the levels of AEA decrease, it triggers the release of 2-AG, which inhibits the hypothalamic-pituitary-adrenal (HPA) axis. This helps prevent overactivation of the axis and maintains a healthy stress response [103, 121]. During stress recovery, decreased levels of PEA and OEA could be linked to changes in inflammatory parameters [66]. PEA and OEA activate the peroxisome proliferator-activated receptor Alpha (PPAR-a), which helps reduce inflammation and the expression of pro-inflammatory cytokines [122].

Future studies should use multiple baseline measures and track stress-related trajectories of peripheral endocannabinoids. Participating in experimental stress paradigms can be a reliable method to assess the inducibility of these chemical compounds.

### Endocannabinoids and gender differences

Among all the studies included in this review, only three [45, 58, 59] showed gender-based differences in eCBs, with conflicting results. Studies on humans have largely confirmed the influence of sex and sexual hormones on endocannabinoid activity [123]. Female migraine sufferers had higher FAAH protein, suggesting lower endocannabinoid levels [124]. Men had higher CB1R binding in the limbic system and endocannabinoid tracer reuptake than females [125, 126]. Plasma 2-AG concentrations were 20% higher in males than in females [127]. Another recent study [128] revealed higher levels of 2-AG, AEA, OEA and PEA in males and suggested that eCBs display sexual dimorphism in age ranges corresponding to female pregnancy, menopause, and post-menopause, while male eCBs changes throughout the lifespan are most likely influenced by testosterone levels.

Anandamide have been positively correlated with oestrogen [129]. Plasma AEA levels were highest during ovulation and lowest during the late luteal phase in women with natural menstrual cycles [130] and in endometriosis patients [131]. Decreased oestrogen levels can reduce endocannabinoid signalling [132], which is crucial for negative feedback and can impede HPA initiation [133].

Most selected studies in this review did not consider gender differences, and the sample size was insufficient for data analysis. Further research with demographically balanced samples of sufficient size is required to determine whether changes in peripheral endocannabinoids are similar across both males and females.

### Peripheral endocannabinoids

Preclinical studies suggest that eCB changes in the brain are closely linked to the peripheral nervous system, as indicated by levels of 2-AG [134, 135]. Our systematic review showed that humans with major depressive disorder or alcohol use disorder might have altered circulating levels of eCBs, which are connected to known variables [58, 59, 65]. This may indicate a potential link between the central nervous system (CNS) and peripheral eCBs [136].

The biological significance of peripheral levels of eCBs is not entirely understood. However, it is known that peripheral endocannabinoids can cross the blood-brain barrier (BBB) through the membranes of the brain microvessels’ endothelial cells [137]. It is worth noting that the brain is the main source of eCBs, but peripheral organs such as the liver, gut, fat tissue, and endothelium can also produce and release eCBs. Therefore, changes in plasma endocannabinoid levels may be linked to peripheral symptoms associated with depression, such as metabolic and immune alterations [138]. It is unclear whether these effects are mediated by the CB1R or other signalling pathways, such as the PPAR-a [100].

Although there are strong correlations between brain FAAH levels and eCB levels in the peripheral circulation [45], cerebrospinal AEA levels have not always been found to correspond to serum levels [139] or symptom improvement [140]. Furthermore, increased plasma levels of FAAH substrates could be due to low FAAH in peripheral organs like the liver or a mechanism unrelated to FAAH. Therefore, it is currently unclear if the levels of circulating eCBs are reflective of endocannabinoid levels in the brain [68]. To better understand this correlation, further studies are required to examine both CNS and peripheral eCB levels.

### Endocannabinoids and exogenous cannabinoids use

Repeated cannabis use is linked to various neuroadaptations in the ECS [141, 142]; however, there is a lack of research on peripheral endocannabinoids and exogenous cannabinoids use.

A study found that a single intravenous dose of tetrahydrocannabinol (THC) increased plasma levels of 2-AG and AEA, followed by a reduction after 5 h. Oral THC administration also increased 2-AG and AEA levels [143]. In a recent study, smoking cannabis led to increasing levels of THC in the blood but did not significantly change AEA and 2-AG peripheral levels. Higher baseline AEA levels were associated with greater intoxication from cannabis, while heavier cannabis use was linked to lower baseline 2-AG levels [144]. Further studies are needed to examine changes in peripheral endocannabinoids in response to specific active exogenous cannabinoids.

### Limitations

The limitations of this review call for cautious interpretation of the findings. First, only articles written in English were selected, which could exclude valuable information provided by articles in other languages. Moreover, several reviewed studies did not consider differences in gender, age, associated comorbidities, or antidepressant treatment. Additionally, endocannabinoid measures were taken during different phases of depressive disorder and length of abstinence in AUD patients. Furthermore, there is evidence that circulating 2-AG concentrations rise significantly between 7 and 11 a.m [145]. , whereas the concentrations of AEA do not change significantly, except following the morning meal [146]. These findings could contribute to the magnitude of the changes in peripheral endocannabinoid levels [73]. Other factors that may have affected our findings include variabilities in diet, including the content, quantity, and frequency of food consumed, which can affect tissue concentrations of eCBs [45].

Given these limitations, larger cohorts are required to achieve sufficient statistical power to consider the influences of relevant covariates. However, eCB levels were often considered secondary or exploratory in the selected studies. Although the findings are informative, they may be at an elevated risk of type I and/or II error due to multiple testing, statistical power, and study design. Another significant challenge in future studies is replicating the available results by applying similar procedures. Clinical studies that employ minimally invasive techniques such as neuroimaging and utilize accessible biological samples like blood are crucial. With specific selection criteria, these studies can further explore how endocannabinoids (eCBs) could serve as potential biomarkers for diagnosis, prognosis, and therapeutic targets in MDD and AUD.

## Electronic supplementary material

Below is the link to the electronic supplementary material.

Supplementary Material 1

## Data Availability

All data generated or analysed during this study are included in this published article [and its supplementary information files].

## Abbreviations

ECS: Endocannabinoid system
eCB: Endocannabinoid
HCV: Hepatitis C virus
DSM-V: Diagnostic and statistical manual of mental disorders
SCID: Structured Clinical Interview for DSM Disorders
HAM-D: Hamilton Depression Rating Scale
BDI: Beck Depression Inventory
ICG: Inventory of Complicated Grief
MDD: Major depressive disorder
AUD: Alcohol Use Disorder BMI, body mass index
SD: Standard deviation
LC/MS: Liquid chromatography-mass spectrometry
LC/MS/MS: Liquid chromatography with tandem mass spectrometry
AEA: Anandamide
2-AG: 2-arachidonyl-glycerol
OEA: Oleoyl-ethanolamide
FAAH: Fatty acid amide hydrolase
EPEA: Eicosapentaenoylethanolamide
PEA: Palmitoylethanolamide
DGLEA: Dihomo-gamma-linolenoyl ethanolamide
DEA: Docosatetraenoyl ethanolamide
DHEA: Docosahexaenoyl ethanolamine
POEA: Palmitoleoyl ethanolamide
