# Supplementary material for: Peripheral endocannabinoids in major depressive disorder and alcohol use disorder: a systematic review
Source: BMC Psychiatry. 2024 Aug 8;24:551. doi: 10.1186/s12888-024-05986-8 (PMC11308641; doi:10.1186/s12888-024-05986-8)
Supplement: Supplementary file 1 — Supplementary Material 1 [file 12888_2024_5986_MOESM1_ESM.docx]

**Supplementary**

**Peripheral Endocannabinoids in Major Depressive Disorder and Alcohol Use Disorder: A systematic review.**

J.J. Fuentes, J. Mayans, M. Guarro, I. Canosa, J. Mestre, F. Fonseca, M. Torrens

**Contents**

**Complete search strategy**

**Table 1.** Quality assessment of studies in detail (ROBINS-I)

**Table 2.** Quality assessment of studies in detail (ROB-2)

**Table 3.** Quality assessment of studies in detail (NOS): Cohort studies

**Table 4.** Quality assessment of studies in detail (NOS): Cross-sectional studies

**Complete search strategy**

Medline

("depressive disorder"[MeSH Terms] OR "depression"[MeSH Terms] OR "depressive disorder"[Tw] OR "depressive disorders"[Tw] OR "depression"[Tw] OR "alcohol abstinence"[MeSH Terms] OR "alcohol drinking"[MeSH Terms] OR "alcohol withdrawal"[Tw] OR "alcohol abuse"[Tw] OR "alcohol addiction"[Tw] OR "alcoholic intoxication"[Tw] OR "alcohol-related disorders"[MeSH Terms] OR "alcohol use disorder"[Tw] OR "alcoholism"[Tw]) AND ("cannabinoids"[MeSH Terms] OR "endocannabinoids"[MeSH Terms] OR "cannabinoid receptor modulators"[MeSH Terms] OR "endocannabinoids"[Tw] OR "endocannabinoid"[Tw] OR "stearoylethanolamide"[Tw] OR "stearoyl ethanolamide"[Tw] OR "palmitoylethanolamide"[Tw] OR "palmitoyl ethanolamide"[Tw] OR "oleoylethanolamide"[Tw] OR "oleoyl ethanolamide"[Tw] OR "palmitoleoylethanolamine"[Tw] OR "anandamide"[Tw] OR "arachidonoylethanolamine"[Tw] OR "arachidonoyl ethanolamine"[Tw] OR "n-acylethanolamine"[Tw] OR "linoleoylethanolamide"[Tw] OR "linoleoyl ethanolamide"[Tw] OR "docosahexaenoylethanolamide"[Tw] OR "docosahexaenoyl ethanolamide"[Tw] OR "2-arachidonoylglycerol"[Tw] OR "2-arachidonoyl glycerol"[Tw] OR "2-linoleoylglycerol"[Tw] OR "2-linoleoyl glycerol"[Tw])

Web of Science

(TI=("Depressive disorder" OR "Depressive disorder*" OR "Depressive disorders" OR "Depressive disorder*" OR "Depression" OR "Depression*" OR "Alcohol use disorder" OR "Alcohol use disorder*" OR "alcohol abstinence" OR "Alcohol abstinence*" OR "alcohol addiction" OR "alcohol addiction*" OR "alcoholism" OR "alcoholism*" OR "alcohol abuse" OR "alcohol abuse*" OR "alcohol drinking" OR "alcohol drinking*") OR AK=("Depressive disorder" OR "Depressive disorder*" OR "Depressive disorders" OR "Depressive disorder*" OR "Depression" OR "Depression*" OR "Alcohol use disorder" OR "Alcohol use disorder*" OR "alcohol abstinence" OR "Alcohol abstinence*" OR "alcohol addiction" OR "alcohol addiction*" OR "alcoholism" OR "alcoholism*" OR "alcohol abuse" OR "alcohol abuse*" OR "alcohol drinking" OR "alcohol drinking*") OR AB=("Depressive disorder" OR "Depressive disorder*" OR "Depressive disorders" OR "Depressive disorder*" OR "Depression" OR "Depression*" OR "Alcohol use disorder" OR "Alcohol use disorder*" OR "alcohol abstinence" OR "Alcohol abstinence*" OR "alcohol addiction" OR "alcohol addiction*" OR "alcoholism" OR "alcoholism*" OR "alcohol abuse" OR "alcohol abuse*" OR "alcohol drinking" OR "alcohol drinking*")) AND (TI=("cannabinoid" OR "cannabinoid*" OR "cannabinoids" OR "cannabinoids*" OR "endocannabinoid" OR "endocannabinoid*" OR "endocannabinoids" OR "endocannabinoids*" OR "cannabinoid receptor modulator" OR "cannabinoid receptor modulator*" OR "cannabinoid receptor modulators" OR "cannabinoid receptor modulators*" OR "stearoylethanolamide" OR "stearoyl ethanolamide" OR ”palmitoylethanolamide" OR "palmitoyl ethanolamide" OR "oleoylethanolamide" OR "oleoyl ethanolamide" OR "palmitoleoylethanolamine" OR "anandamide" OR "arachidonoylethanolamine" OR "arachidonoyl ethanolamine" OR "n-acylethanolamine" OR "linoleoylethanolamide" OR "linoleoyl ethanolamide" OR "docosahexaenoylethanolamide" OR "docosahexaenoyl ethanolamide" OR "2-arachidonoylglycerol" OR "2-arachidonoyl glycerol" OR "2-linoleoylglycerol" OR "2-linoleoyl glycerol") OR AK=("cannabinoid" OR "cannabinoid*" OR "cannabinoids" OR "cannabinoids*" OR "endocannabinoid" OR "endocannabinoid*" OR "endocannabinoids" OR "endocannabinoids*" OR "cannabinoid receptor modulator" OR "cannabinoid receptor modulator*" OR "cannabinoid receptor modulators" OR "cannabinoid receptor modulators*" OR "stearoylethanolamide" OR "stearoyl ethanolamide" OR ”palmitoylethanolamide" OR "palmitoyl ethanolamide" OR "oleoylethanolamide" OR "oleoyl ethanolamide" OR "palmitoleoylethanolamine" OR "anandamide" OR "arachidonoylethanolamine" OR "arachidonoyl ethanolamine" OR "n-acylethanolamine" OR "linoleoylethanolamide" OR "linoleoyl ethanolamide" OR "docosahexaenoylethanolamide" OR "docosahexaenoyl ethanolamide" OR "2-arachidonoylglycerol" OR "2-arachidonoyl glycerol" OR "2-linoleoylglycerol" OR "2-linoleoyl glycerol") OR AB=("cannabinoid" OR "cannabinoid*" OR "cannabinoids" OR "cannabinoids*" OR "endocannabinoid" OR "endocannabinoid*" OR "endocannabinoids" OR "endocannabinoids*" OR "cannabinoid receptor modulator" OR "cannabinoid receptor modulator*" OR "cannabinoid receptor modulators" OR "cannabinoid receptor modulators*" OR "stearoylethanolamide" OR "stearoyl ethanolamide" OR ”palmitoylethanolamide" OR "palmitoyl ethanolamide" OR "oleoylethanolamide" OR "oleoyl ethanolamide" OR "palmitoleoylethanolamine" OR "anandamide" OR "arachidonoylethanolamine" OR "arachidonoyl ethanolamine" OR "n-acylethanolamine" OR "linoleoylethanolamide" OR "linoleoyl ethanolamide" OR "docosahexaenoylethanolamide" OR "docosahexaenoyl ethanolamide" OR "2-arachidonoylglycerol" OR "2-arachidonoyl glycerol" OR "2-linoleoylglycerol" OR "2-linoleoyl glycerol"))

Embase

((exp *"Depressive disorder"/ OR "Depressive disorder*".ti,ab OR "Depressive disorders".ti,ab OR "Depressive disorder*".ti,ab OR "Depression".ti,ab OR "Depression*".ti,ab OR exp *"alcohol abstinence"/ OR "Alcohol use disorder".ti,ab OR "Alcohol use disorders*".ti,ab "Alcoholism".ti,ab OR "Alcohol abuse*".ti,ab OR "alcohol drinking".ti,ab OR "alcoholism*".ti,ab OR "alcohol use disorder*".ti,ab OR ”alcohol addiction”.ti,ab OR ”alcohol withdrawal”.ti,ab) AND ("cannabinoid".ti,ab OR "endocannabinoid*".ti,ab OR "cannabinoids".ti,ab OR "endocannabinoids*".ti,ab OR "cannabinoid receptor modulator".ti,ab OR exp *"stearoylethanolamide"/ OR "stearoyl ethanolamide".ti,ab OR exp *”palmitoylethanolamide"/OR exp *"oleoylethanolamide"/ OR "oleoyl ethanolamide".ti,ab OR "palmitoleoylethanolamine".ti,ab OR "anandamide".ti,ab OR "arachidonoylethanolamine".ti,ab OR exp *"arachidonoyl ethanolamine"/ OR "n-acylethanolamine".ti,ab OR "linoleoylethanolamide".ti,ab OR exp *"linoleoyl ethanolamide"/ OR exp *"docosahexaenoylethanolamide"/ OR "docosahexaenoyl ethanolamide".ti,ab OR "2-arachidonoylglycerol*".ti,ab OR exp "2-arachidonoyl glycerol"/ OR exp *"2-linoleoylglycerol"/ OR "2-linoleoyl glycerol".ti,ab)

**Table 1.** Quality assessment of studies in detail (ROBINS-I)

| **Domains** | **Risk of bias** | **Reason** |
| --- | --- | --- |
| **Lazary et al. (2021)** |  |  |
| Confounding | Moderate | We acknowledge that optimal assessment of confounding risk is not possible in a single-arm clinical trial. Certain important potential confounders were assessed (e.g., BMI). |
| Selection of patients | Low | All patients who would have been eligible for the target trial were included in the study and start of intervention and follow-up coincided. |
| Classification of interventions | Low | The intervention group was clearly defined and classification of intervention status was solely based on information collected at the time of intervention. |
| Deviations from intended interventions | Low | Deviations from intended intervention reflected usual practice and were unlikely to impact the outcome. |
| Missing data | Low | Data was reasonably complete and missing data was addressed appropriately. |
| Measurement of outcomes | Moderate | Patients and people delivering the interventions were aware of the patients’ assigned intervention during the trial. The primary outcome was assessed with well described and objective measures. |
| Selection of reported results | Low | There is clear evidence that reported results correspond to the intended outcomes. |
| Overall risk of bias | Moderate | Moderate risk of bias with confounding and measurement of outcomes, meaning overall risk of bias is classified as moderate risk of bias. |
|  |  |  |
| **Meyer et al. (2019)** |  |  |
| Confounding | Serious | We acknowledge that optimal assessment of confounding risk is not possible in a single-arm clinical trial. No potential confounders were assessed. Participants self-reported significantly lower depressive symptoms and anxiety for the preferred exercise session than moderate session. Precise control of substance was not performed. |
| Selection of patients | Low | All patients who would have been eligible for the target trial were included in the study and start of intervention and follow-up coincided. |
| Classification of interventions | Moderate | The intervention group was clearly defined but individual choices for the preferred exercise session may have limited statistical power. |
| Deviations from intended interventions | Low | Deviations from intended intervention reflected usual practice and were unlikely to impact the outcome. |
| Missing data | Low | Data was reasonably complete and missing data was addressed appropriately. |
| Measurement of outcomes | Moderate | Patients and people delivering the interventions were aware of the patients’ assigned intervention during the trial. The primary outcome was assessed with self-reported measures. |
| Selection of reported results | Moderate | There is clear evidence that reported results correspond to the intended outcomes but are secondary analyses from a larger sample. |
| Overall risk of bias | Serious | Serious risk of bias with confounding, moderate risk with classification of interventions and measurement of outcomes and some concerns with selection of reported results, meaning overall risk of bias is classified as serious risk of bias. |

**Table 2.** Quality assessment of studies in detail (ROB-2)

| **Domains** | **Risk of bias** | **Reason** |
| --- | --- | --- |
| **Yang et al. (2019)** |  |  |
| Randomization process | Low | Allocation sequence was concealed and random with no suggested differences between the groups, indicating no issues with randomization process. |
| Deviations from intended interventions | Low | There were no deviations from the intended intervention. |
| Missing outcome data | Some concerns | One participant from the EPA group and two from the DHA group did not provide baseline and 12-week plasma measures. |
| Measurement of the outcome | Low | Method of measuring the outcome was appropriate and measurement or ascertainment of the outcome did not differed between the intervention groups. Outcome assessors were not aware of the intervention received by study participants. |
| Selection of the reported results | Low | Reported primary outcome was in accordance with the pre-specified analysis plan in the protocol, was unlikely to have been selected from multiple eligible outcome measurements within the outcome domain and unlikely to have been selected on the basis of the results. |
| Overall risk of bias | Some concerns | Some concerns with missing outcome data, while all other domains had a low risk, meaning overall risk of bias is classified as some concerns. |
|  |  |  |
| **Brellenthin et al. (2019)** |  |  |
| Randomization process | Some concerns | Allocation sequence was concealed and random with no suggested differences between the groups, but a not described online service was used. |
| Deviations from intended interventions | Low | There were no deviations from the intended intervention. |
| Missing outcome data | Moderate | Three individuals did not attend more than their baseline visit and were not included in within-sessions. The eight participants who began exercise completed on average 15 out 18 (83%) of their scheduled exercise visits. |
| Measurement of the outcome | Some concerns | Method of measuring the outcome was appropriate and measurement or ascertainment of the outcome did not differed between the intervention groups. Outcome assessors were aware of the intervention received by study participants, but they used objective and validated tools. |
| Selection of the reported results | Low | Reported primary outcome was in accordance with the pre-specified analysis plan in the protocol, was unlikely to have been selected from multiple eligible outcome measurements within the outcome domain and unlikely to have been selected on the basis of the results. |
| Overall risk of bias | Moderate | Some concerns with missing outcome data and measurement of the outcome. Moderate risk in missing outcome data, meaning overall risk of bias is classified as moderate. |

**Table 3.** Quality assessment of studies in detail (NOS): Cohort studies

| **Study** | Selection of cohorts | | | | Comparability of cohorts | Outcome | | |  |
| --- | --- | --- | --- | --- | --- | --- | --- | --- | --- |
|  | **Representativeness of the exposed cohort** | **Selection of the non exposed cohort** | **Ascertainment of exposure** | **Demonstration that outcome of interest was not present at start of study** | **Comparability of cohorts on the basis of the design or analysis** | **Assessment of outcome** | **Was follow-up long enough for outcomes to occur** | **Adequacy of follow up of cohorts** | **Total score** |
| **Hill et al. 2009** | - | + | + | + | + | + | - | + | 6 / Good quality |
| **Mangieri et al. 2009** | - | - | + | + | - | + | - | + | 4 / Poor quality |
| **Best et al. 2020** | - | - | + | + | + | + | + | - | 5 / Fair quality |
| **Bersani et al. 2021** | - | - | + | + | ++ | + | + | - | 6 / Fair quality |
| **Kang et al., 2021 (USA)** | - | + | + | + | + | + | + | + | 7 / Good quality |
| **Zajkowska et al., 2020 (UK)** | - | - | + | + | + | + | + | - | 5 / Fair quality |
| **Spagnolo et al., 2016 (USA)** | - | + | + | + | ++ | + | + | + | 8 / Good quality |

**Table 4.** Quality assessment of studies in detail (NOS): Cross-sectional studies

| **Study** | **Selection** | | | | **Comparability** | **Outcome** | |  |
| --- | --- | --- | --- | --- | --- | --- | --- | --- |
|  | **Representativeness of the sample** | **Sample size** | **Non-respondents** | **Ascertainment of the exposure (risk factor)** | **Comparability** | **Assessment of the outcome** | **Statistical test** | **Total score** |
| **Romero- Sanchiz et al. 2019** | + | - | - | + | + | ++ | + | 6 / Fair quality |
| **Hill et al. 2008** | - | - | - | + | + | ++ | + | 5 / Poor quality |
| **Coccaro et al. 2018** | + | - | - | + | - | + | + | 5 / Poor quality |
| **Garcia-Marchena et al. 2017** | + | - | - | ++ | + | + | + | 6 / Fair quality |
| **Harfmann et al. 2020** | - | - | - | + | + | ++ | + | 5 / Poor quality |
| **Behnke et al. 2022** | - | - | - | + | + | ++ | + | 5 / Poor quality |
